# Smear positive pulmonary tuberculosis and HIV co-infection in prison settings of North Gondar Zone, Northwest Ethiopia

**DOI:** 10.1186/s12889-016-3761-y

**Published:** 2016-10-18

**Authors:** Teklay Gebrecherkos, Baye Gelaw, Belay Tessema

**Affiliations:** Department of Medical Microbiology, School of Biomedical and Laboratory sciences, College of Medicine and Health Sciences (CMHS), University of Gondar (UOG), P.O. box 196, Gondar, Ethiopia

**Keywords:** Prison, Pulmonary tuberculosis, HIV, Malnutrition, Poor ventilation, TB contact

## Abstract

**Background:**

In correctional settings tuberculosis is a public health concern. The incarcerated population is at greater risk for tuberculosis (TB) than the general population. The aim of this study was to determine the prevalence of smear positive pulmonary tuberculosis (PTB) and associated risk factors in prison settings.

**Methods:**

A cross-sectional study was conducted among prisoners of North Gondar zone where all inmates with a history of cough for ≥ 2 weeks were included. Socio-demographic characteristics and potential risk factors were assessed using a structured questionnaire. Spot-morning-spot sputum samples were collected, smears were prepared and stained with Auramine O stain and examined through light emitting diode- fluorescence microscope. All samples positive for acid-fast bacilli were further examined by GeneXpert MTB/RIF assay. Data was analyzed using SPSS version 20 and a *P*-value < 0.05 was taken as statistically significant. The multivariable logistic regression analysis was used to determine the association between risk factors and prison tuberculosis.

**Results:**

A total of 282 prison inmates suspected of PTB were enrolled in the study. The overall prevalence of smear positive PTB infection was 5.3 % (15/282), but none of the smear positive TB cases were resistant to rifampicin. The prevalence of HIV infection among TB suspected prisoners and smear positive PTB cases was 6 and 27 %, respectively. Moreover, smear positive PTB disease was significantly associated with smoking status, malnutrition, number of prison inmates per cell, poor cell ventilation, and a history of contact with TB patients.

**Conclusion:**

The prevalence of smear positive pulmonary tuberculosis among north Gondar prison inmates was still high although lower than previous reports. There was a high prevalence of HIV among smear positive PTB cases. Reducing the burden of prison inmates in a particular cell, preventing malnutrition, establishing ventilation system can possibly minimize the transmission of tuberculosis among prisoners.

## Background

Tuberculosis (TB) is an air borne infectious disease caused by the bacillus *Mycobacterium tuberculosis*. It typically affects the lungs (pulmonary TB), but it can also affect other sites (extra pulmonary TB). The disease spreads in the air when sick people expel the bacteria while talking, coughing, singing, sneezing and spitting [1]. Once a person develops pulmonary tuberculosis (PTB), several suggestive clinical presentations, such as cough for more than 2 weeks, production of sputum and weight loss together with other respiratory symptoms, like chest pain, haemoptysis, breathlessness and/or constitutional symptoms, like night sweats, fever, loss of appetite, and fatigue can also occur [2].

Prisons act as reservoirs for TB, pumping the disease into the civilian community through staff, visitors, and inadequately treated former inmates [3]. Despite the fact that the global focus on TB control is on early diagnosis and treatment of people in high TB and TB/HIV-endemic countries, people in prisons are often neglected reservoirs for TB transmission, threatening those in the outside community [2]. Tuberculosis in prison settings poses a major public health problem worldwide. Prisons are settings in which TB transmission occurs and high rates of active TB have been reported worldwide, especially in countries in the former Soviet Union [4] and in Sub-Saharan Africa (SSA) [5], where TB in prisons threatens not only prison inmates, but also prison staff who eventually interact directly with their families and the community after work [6]. This indicates that TB in prisons is the concern of not only prisoners but also of the wider society [7]. According to the World Health Organization (WHO) 2013 report, the prevalence of TB in prisons is very high, accounting for up to 25 % of the TB burden in a given country, and is estimated to be 10 to 100 folds higher than among the general population [8]. High levels (60.0 %) of MDR-TB were reported from prisons in Belarus earlier [9]. Prisons are increasingly becoming the ideal breeding grounds for the concentration and dissemination of TB, including MDR-TB, from which infection is transmitted to the general population [10].

Prisoners constitute a high risk group for the acquisition of *Mycobacterium tuberculosis* infection compared with the general population, due to overcrowding, poor ventilation, low socioeconomic status, poor nutrition, and poor health condition of prison inmates which can predispose them to a high risk of TB incidence [11]. Prison inmates are often highly mobile, rotating from cell to cell and from prison to prison for another incarceration, and they may be released after some time and may contribute to tuberculosis transmission [12]. Studies also showed that malnutrition was associated with increased risk of developing TB in prisons. For instance, a study in Zambia found that nutritional status and food intake were universally poor in all surveyed prisons [13].

According to previous reports, the prevalence of TB was high in prisons in Africa, with the actual magnitude varying from country to country. Thus, a prevalence of 3, 574 per 100,000, 7576 per 100,000 and 6821 per 100,000 was reported in Malawi, Zambia and Madagascar, respectively [14–16]. In Botswana the point prevalence of tuberculosis among prisoners and guards was reported as 3797/100,000 and 2662/100,000, respectively [17]. Previously, prison tuberculosis was also reported in some parts of Ethiopia. Previously, Abebe et al, (2008) conducted a survey in three major prisons in the eastern parts of Ethiopia [18] and found an 8.9 % prevalence of pulmonary tuberculosis among prison inmates. A study conducted in the southern parts of Ethiopia reported a 19.4 % prevalence of pulmonary tuberculosis among prison inmates [19]. Another report from a single prison in Gondar town reported a prevalence of 1482 per 100,000 smear positive pulmonary tuberculosis among prison inmates [20]. Because this report was issued from a single prison in Gondar town, we believe that the report lacked representativeness as there are prisons in different weredas of North Gondar zone. Thus, this study aimed to determine the prevalence of smear positive pulmonary tuberculosis and associated risk factors among prisoners in North Gondar zone, Ethiopia.

## Methods

### Study Design, area and period

A cross-sectional study was conducted on four prisons in North Gondar Administrative Zone to estimate the prevalence of smear positive pulmonary tuberculosis rates among prison inmates. The study was conducted from February to April 2015, at 4 prisons, namely Debark, Dabat, Chilga and Gondar towns of North Gondar zone, northwest Ethiopia. North Gondar zone has 21 weredas with 539 kebeles and 2 town administrations. According to the 2007 Ethiopian census, North Gondar has a total population of 2,929,628 (1,486,040 men and 1,443,588 women). In this zone, there are one zonal and 21 wereda police stations. There are 4 large prisons that could house about 1500–3500 prisoners. They detain mainly sentenced and some pre-trial prisoners from several surrounding weredas.

### Population

The source population for this study was all of the four prison inmates present in North Gondar zone. However, only prison inmates who had cough for ≥ 2 weeks during the study period were recruited and included as the sample population.

### Inclusion/exclusion criteria and study variables

Prison inmates who were willing to participate and had 2 weeks and above duration of cough were included in the study. On the other hand, prison inmates who had 2 weeks and above duration of cough but were unable to produce sputum, and prison inmates who were on anti-TB treatment and/or provided incomplete information were excluded from the study. The prevalence of smear positive pulmonary tuberculosis was used as the dependant variable whereas age, sex, smoking status, educational status, residence, marital status, incarceration time, history of previous contact with active TB cases, frequency of imprisonment, nutritional status, number of prisoners per cell, sharing food and other materials, pervious treatment for tuberculosis, window opening practice, HIV status, occupation, and duration of cough were used as independent variables.

### Sample size and sampling technique

The sample size was determined using the following single population proportion formula: N = z^2^p (1 − p)/w^2^, where N = the number of TB suspected prison inmates, Z = standard normal distribution value at 95 % CI which is 1.96, P = the prevalence of pulmonary tuberculosis among prison inmates (10.4 %, previous prevalence report from Gondar prison), W = the margin of error, taken as 4 %. Accordingly, the sample size calculated was 223. When the record of prisons was reviewed, the average prison inmates in the 4 prisons of north Gondar were found to be 3900. As this source population is less than ten thousand, the sample size was corrected using the formula nf = in/1 + in/N, where nf is the final sample size (223) and N the source population. Considering a 10 % no response rates the final sample size of 235 was determined, but all inmates who fulfilled the inclusion criteria were enrolled and the final sample size was 282.

At the time of the study about 3900 inmates were detained in the four prisons of the north Gondar zone. A mass screening strategy was used to identify PTB suspects. This strategy provides an equal chance of selecting eligible individuals and reduces the chance of losing PTB suspects. First, all prisoners were collectively questioned about the presence of cough; then prisoners with cough were individually interviewed about the duration of the cough. Prisoners who had a cough history of ≥ 2 weeks were included in the study. In this case, a total of 282 prisoners were selected to participate in the study.

### Socio-demographic data collection

Once eligible inmates were recruited for the study, socio-demographic characteristics were collected. Information on socio-demographic characteristics, imprisonment, number of prisoners per cell, history of previous treatment for TB, previous exposure to TB, window opening practice, cigarette smoking, and others were collected using a structured and pretested questionnaire.

### Sputum sample collection, processing and staining

Spot-morning-spot sputum samples were collected following Standard Operation Procedures (SOPs) of the University of Gondar Hospital Laboratory. Briefly, the sputum samples were collected using dry, clean, leak proof, translucent, and screw-capped plastic containers with a capacity of 30 ml. The sputum samples were collected by the principal investigator from each participant, and the sample was transported to the College of Medicine and Health Sciences (GCMHS) Microbiology Laboratory for processing and examination. Smear was prepared by taking a portion of the purulent part of the sputum sample on a glass slide. The sample was spread on a glass slide and allowed to air dry, and then heat-fixed by passing the preparation through a flame 2 to 3 times. Each smear was stained with Auramine O staining procedure. Briefly, 0.1 % Auramine O solution was flooded on the smears and allowed to stain for 20 min. The stain was rinsed with water, drained and decolorized with 0.5 % acid-alcohol for 3 min. The preparation was rinsed with water until the macroscopically visible stain was washed away and drained. The smears were flooded with 0.5 % potassium permanganate solution for 1 min to minimize non-specific fluorescence and washed with water followed by air dry and microscopic examination. Stained slides were examined under 20x, and 40x magnifications of Primo Star iLED, light emitting diode (LED) fluorescence microscopy (FM) for AFB. The AFB appeared bright yellow against dark back ground materials. All AFB positive samples were also re-examined by staining another batch of smears with Ziehl-Neelsen staining method. Briefly, smears were flooded with carbonfuchsin solution and heated from the underneath until steaming and allowed to stand for 5 min. After washing with tap water, smears were decolorized with acid alcohol for 1 min and washed with tap water and counter-stained with methylene blue for 30 s, washed and air dried. Slides were examined under 100x oil immersion objective with bright field illumination.

### GeneXpert MTB/RIF assay

All AFB positive sputum samples were further examined using the GeneXpert MTB/RIF (Cepheid Gene Xpert system) procedure to detect rifampicin (RIF) resistance. In brief, sample reagent was added to a sputum sample in a 2:1 (V/V) proportion and mixed by hand shaking. The preparation was incubated at room temperature for 15 min. Two ml of the treated sputum sample was transferred to the cartridge using a sterile pipette and the GeneXpert MTB/RIF machine run was initiated. After about 2 h, the result was interpreted and displayed by the machine as MTBC detected/not-detected and “RIF resistance detected or not detected”.

### HIV screening

In this study, all recruited participants were screened for HIV infection after getting consent from each inmate. Concerning HIV testing, nurses working at the Providing Initiative Counseling and Testing (PICT) clinics of the prison were involved. Prisoners with positive sputum smears were placed on an anti-tuberculosis treatment as recommended by the National Tuberculosis and Leprosy Control Program (NTLCP) guidelines [21], while smear-negative patients were given a 10-day course of broad spectrum antibiotic treatment.

### Nutritional assessment

Body weight was determined to the nearest 0.1 kg using an electronic digital scale, and height was measured to the nearest 0.1 cm. Body mass index (BMI) was used to determine the nutritional status of the prisoners as under nutrition (BMI < 18.5 kg/m^2^) and normal (BMI = >18.5 kg/m^2^) [22].

### Quality control

Data quality was maintained using a questionnaire translated from English to Amharic. Pre-testing of the questionnaire was done for completeness and appropriateness before data collection. The reliability of the findings was guaranteed by implementing quality control measures throughout the whole processes of the laboratory work. To assure the quality of the sputum specimen, proper sputum collection, handling and processing procedures were used. The quality of newly prepared reagents was determined by both positive and negative slides. The performance of the microscopy was checked by both negative and positive stained slides. All slides were seen by the principal investigator and senior laboratory workers after staining all preparations with Auramine O stain. To check the accuracy, Auramine O positive slides were also re-examined by preparing corresponding smear and stained with Ziehl-Neelsen stain. Moreover, discordant results between Auramine O and Ziehl-Neelsen microscopy were subjected to GeneXpert procedures.

### Data processing and analysis

Data was checked for completeness, cleaned manually, and entered and analyzed using the Statistical Package for the Social Sciences (SPSS) version 20. All variables of the study were initially tested for association with smear positivity by using the binary logistic regression model. Variables which showed statistically significant association with smear positivity by the binary logistic regression model were put into the multivariable analysis model to check if the association existed after controlling against all of the rest of the variables, and a *P*-value less than 0.05 was considered as statistically significant.

## Results

A total of 3900 prison inmates were found in the four prisons of North Gondar Administrative Zone. The inmates were 580, 350, 700, and 2270 for Debark, Dabat, Chilga and Gondar town prisons, respectively. However, only 282 prisoners were eligible because they had cough for ≥ 2 weeks. The numbers of pulmonary TB suspected cases were 48, 30, 50, and 154 but the proportion of smear positive pulmonary tuberculosis cases were 6.25 % (3/48), 0 % (0/30), 2 % (1/50), and 7.1 % (11/154), respectively. The majority (80.2 %) developed the cough after imprisonment. On the other hand, 9.6 % had a history of previous treatment for TB, and 1.1 % of them were positive for acid-fast bacilli at the moment.

### Socio-demographic characteristics

Among the 282 TB suspects, 98.2 % were male, 68.4 % married, and the other 27.3 % single. The educational status tally showed that 50 % were unable to read and write, while 12.1 % attended high school and above. The majority (78 %) of the TB suspects were rural dwellers. The mean age was 35 years (SD ± 11.9) and the median age 30 (IQR: 16 to 80) years. The proportion of farmers was 78.7 %, followed by students (12.1 %) (Table 1).

**Table 1:** Socio-demographic characteristics of tuberculosis suspected prison inmates in North Gondar Zone Prisons, February—April 2015 (*n* = 282)

Variables	Sputum Microscopy result						
	Smear + ve n (%)	Smear -ve n (%)	Total	COR (95 % Cl)	*P*-Value	Adjusted OR (95 % Cl)	*P*-value
Age							
15–24	3 (20)	60 (22.3)	63 (22.3)	1		1	1
25–34	6 (40)	93 (35)	99 (35.1)	1.29 (0.31–5.35)	0.72	2.33(0.52–13.69)	0.662
34–44	2 (13.3)	48 (18)	50 (17.7)	0.83 (0.13–5.19)	0.84	1.45(0.16–13.23)	0.655
> 45	4 (26.7)	66 (24.7)	70 (24.8)	1.21 (0.26–5.63)	0.80	1.53 (0.24–12.3)	0.890
Sex							
Male	15 (5.3)	262(92.9)	277(98.2)	1		1	1
Female	0(0.0)	5(1.8)	5(1.8)	NC	0.99	1	0.99
Residence							
Urban	2 (13.3)	60 (22.5)	62 (22)	1		1	1
Rural	13(86.7)	207(75.5)	220 (78)	1.88 (0.418.5)	0.413	2.81 (0.38–10.07)	0.317
Education							
No read and write	7(46.7)	134 (50.2)	141(50)	1		1	1
Elementary	6 (40)	101(37.8)	107(37.9)	1.14 (0.37–3.48)	0.820	1.36 (0.38–4.83)	0.217
High school and above	2 (13.3)	32 (12)	34 (12.1)	1.19 (0.24–6.03)	0.828	4.95 (0.39–62.91)	0.297
Occupation							
Civil servant	0 (0)	13 (4.9)	13 (4.6)	1		1	1
Farmer	14(93.3)	208 (77.9)	222(78.7)	1.08 (0.48–7.31)	0.99	NC	0.99
Students	1 (6.7)	33 (12.4)	34 (12.1)	4 (0.8–9.2)	0.99	NC	
Marital status							
Single	5 (33.3)	72 (27)	77 (27.3)	1		1	1
Married	10(66.7)	183 (68.5)	193 (68.4)	0.79 (0.26–2.36)	0.67	0.42 (0.09–1.99)	0.277
Divorced	0(0)	12 (4.5)	12 (4.3)	0	0.99	NC	0.99

*TB* pulmonary tuberculosis, *OR* odds ratio, *CI* Confidence interval, *COR* Crude odds ratio, *NC* Not computed due to small numbers

+ve = positive; -ve = negative

*P ⩽ 0.05 (significance level)

### Prevalence of pulmonary tuberculosis among prisoners

The overall prevalence of smear positive pulmonary tuberculosis among prison inmates that had symptoms of PTB was 5.3 %. In this study, none of the smear positive cases were resistant to refampicin (RIF). The prevalence of smear positive pulmonary tuberculosis among tuberculosis suspected prisoners in the prison of Gondar town (prison with largest number of inmates, *n* = 2270, with 150 tuberculosis suspected cases) was 6.7 %, but 3 % for Dabat prison that had the least number of prison inmates (*n* = 350) and tuberculosis suspected cases (*n* = 33). All the smear positive cases were male, and six out of the 15 smear positive pulmonary tuberculosis cases were in the age group of 25–34 years. The majority of the smear positive TB cases (86.7 %; *n* = 13) were living in rural areas prior to their imprisonment, and seven out of fifteen were illiterate (Table 2).

**Table 2:** Assessment of the prisons environment, incarcerated time and number of prisoner in a particular prison room compared with smear positive pulmonary tuberculosis infection among North Gondar Zone prisons, February—April 2015

Variables	Sputum Microscopy result						
	Smear + ve n (%)	Smear -ve n (%)	Total	COR (95 % Cl)	*P*-Value	Adjusted OR (95 % Cl)	*P*-value
Incarcerated time (Month)							
< 2	2 (13.3)	25 (9.4)	27 (9.6)	1		1	1
2–6	3 (20)	55 (20.6)	58 (20.6)	0.68 (0.11–4.330	0.685	1.54 (0.12–10.30)	0.970
7–12	2 (13.3)	63 (23.6)	65 (23)	0.40 (0.05–2.97)	0.36	0.79 (0.06–5.65)	0.624
> 12	8 (53.4)	124 (46.4)	132 (46.8)	0.81 (0.16–0.53)	0.029	1.02 (0.11–5.26)	0.759
Frequency of imprisonment							
Once	14(93.3)	258(96.6)	272(96.5)	1		1	1
Twice and above	1 (6.7)	9 (3.4)	10 (3.5)	2.05 (0.24–17.31)	0.511	10.52(0.78–142.10)	0.062
Number of prisoners per cell							
< 50	2 (13.3)	55 (20.6)	57 (20)	1		1	1
51–100	1 (6.7)	73 (27.3)	74 (26)	0.38 (0.03–4.26)	0.43	0.19 (0.01–2.90)	0.006
> 100	12 (80)	139(52.1)	151(53.5)	2.37 (0.52–10.95)	0.048	3.32 (3.29–8.51)	0.002*
Window opening practice							
Always	6 (40)	108(40.4)	114 (40.4)	1		1	1
Some times	0 (0)	11 (4.12)	11 (3.9)	NC	0.99	NC	0.202
Never	9 (60)	148(55.5)	157 (5 5.7)	1.09 (0.37–3.17)	0.028	11.28 (2.25–56.7)	0.003*

*TB* pulmonary tuberculosis, *OR* odds ratio, *CI* Confidence interval, *COR* Crude odds ratio, *NC* Not computed due to small numbers

+ve = positive; -ve = negative

*P ⩽ 0.05 (significance level)

### Association between risk factors and pulmonary tuberculosis in prison settings

The majority (91.5 %) of the tuberculosis suspected prisoners was jailed only once, and the incarceration time ranged from as low as 2 months or less to as high as more than12 months. However, there was no significant association between smear positive pulmonary tuberculosis and neither that of incarceration time nor the frequency of imprisonment. The mean number of inmates per cell was 233 (±126.5, 35–600). Moreover, 53.5 % of the prison rooms had more than 100 inmates per cell, and 55.7 % of the inmates were jailed in a room whose window had never been opened. In this study, smear positive pulmonary tuberculosis among prison inmates was significantly associated with the occurrence of overcrowding (>100/cell) in cells and windows that had never been opened (*P* < 0.003). Prison TB suspects that did not opened their cell windows regularly had eleven times more risk (AOR = 11.28, 95 % CI = 2.25–56.7) to acquire TB than the corresponding cases where regular opening of cell windows was practiced. Eighty-five percent of the TB suspects were sharing food items and other materials. Among the smear positive pulmonary tuberculosis cases, 10 (66.7 %) confirmed their sharing of consumables with TB cases in the cells. On the other hand, 85.8 % of the smear negative TB suspected cases also admitted to the sharing of food items and other consumables with TB cases (Table 3).

**Table 3:** History of tuberculosis contact, smoking status and the prevalence of malnutrition among North Gondar Zone prisons, February—April 2015

Variables	Sputum Microscopy result						
	Smear + ve n (%)	Smear-ve n (%)	Total	COR (95 % Cl)	*P*-value	Adjusted OR (95 % Cl)	*P*-value
Smoking							
No	9 (60)	232 (86.9)	241 (85.5)	1	1	1	1
Yes	6 (40)	35 (13.1)	41 (14.5)	4.42(1.48–13.17)	0.008*	7.16 (1.76–29)	0.006*
Previous contact with TB							
No	4(26.7)	182 (68.2)	186 (66)		1	1	1
Yes	11(73.3)	85 (31.8)	96 (34)	5.88(1.48–13.17)	0.003*	5.03(1.05–19.6)	0.035*
Episode of Cough							
Before imprisonment	6 (40)	50 (18.7)	56 (19.8)	1	1	1	1
After imprisonment	9 (60)	217 (81.3)	226 (80.1)	0.35 (0.12–1.02)	0.05	0.58 (0.15–2.20)	0.328
Duration of Cough (in weeks)							
2	2 (13.3)	51 (19.1)	53 (18.8)	1	1	1	1
3	1 (6.7)	29 (10.9)	30 (10.6)	0.88 (0,07–10.12)	0.918	0.55 (0.03–8.88)	0.67
4	2 (13.3)	63 (22.6)	65 (23)	0.81 (0.11–5.95)	0.83	0.57 (0.05–6.05)	0.64
≥ 8	10(66.7)	124 (46.4)	134 (47.5)	2.05 (0.44–9.72)	0.363	1.45 (0.24–8.81)	0.68
Nutritional Status (BMI (kg/m2)							
≥ 18.5	5 (33.3)	238 (89.1)	243 (86.2)	1	1	1	
< 18.5	10(66.7)	29 (10.9)	39 (13.8)	16.41(5.25–51.35)	<0.001*	16.3(3.89–67.96)	<0.001*
History of previous Treatment							
No	12(80)	243 (91)	255 (90.4)	1	1	1	1
Yes	3 (20)	24 (9)	27 (9.6)	2.53 (0.67–9.59)	0.172	0.24 (0.05–1.80)	0.170
HIV Status							
Negative	11(73.3)	254 (95.1)	265 (94)	1	1	1	1
Positive	4 (26.7)	13 (4.9)	17 (6)	7.12(1.99–25.4)	0.003*	7.26 (1.10–33.3)	0.024*

*TB* pulmonary tuberculosis, *OR* odds ratio, *CI* Confidence interval, *COR* Crude odds ratio

+ve = positive; -ve = negative

*P ⩽ 0.05 (significance level)

The proportions of TB suspected prisoner who smoked cigarettes at the moment was 14.4 % (41/285) of which 6 (14.6 %) were positive for acid-fast bacilli, but the other 241 inmates were non-smokers. Cigarette smoking was significantly associated with smear positive pulmonary tuberculosis rates (*p* = 0.006), and TB suspects who reported cigarette smoking were seven times (AOR = 7.16 and 16.26, 95 % CI = 1.76–29.01) more likely to develop smear positive pulmonary tuberculosis than those who did not smoke. A total of 96 (34 %) TB suspects had a previous history of contact with TB patients and 11 (11.6 %) of these were positive for acid-fast bacilli. Fifty-six prisoners had a history of cough before imprisonment (6 smear positive and 50 smear negative), but the other 226 (9 smear positive and 217 smear negative) developed cough after imprisonment. In this study, pulmonary tuberculosis disease among TB suspects in prison was significantly associated with the history of contact with TB patients (*P* < 0.03). Furthermore, TB suspects who had contact with active TB patients in their vicinity were about five times [AOR = 5.03, 95 % CI = 1.05–19.30) more likely to develop smear positive pulmonary tuberculosis than those who had no history of contact with known tuberculosis patients (Table 3).

The nutritional status of the TB suspects showed that 39 out of the 282 inmates were malnourished (BMI < 18.5 kg/m^2^) making a 14 % prevalence of malnutrition among tuberculosis suspected prison inmates in Gondar. Among the smear positive TB cases, 10 out of 15 were malnourished. Moreover, malnutrition was significantly associated with smear positive pulmonary tuberculosis among tuberculosis suspected prison inmates (*P* < 0.001). Tuberculosis suspects who had malnutrition (BMI <18.5 kg/m2) were sixteen times [AOR = 16.26, 95 % CI = 4.5–67.9) more likely to develop smear positive pulmonary tuberculosis than those who had normal nutritional status.

### HIV-Tuberculosis co-infection

The overall prevalence of HIV infection among TB suspected prison inmates in North Gondar was 6.03 %. The prevalence of HIV infection among smear positive TB cases was 27 % (*n* = 4/15) but 4.9 % (*n* = 13/267) among smear negative prisoners. In this study, HIV sero-prevalence was significantly associated with smear positive pulmonary tuberculosis among TB suspected prisoners (*P* = 0.024; AOR =7.26, 95 % CI = 1.10 – 33.30).

## Discussion

Prisons are considered as reservoirs for facilitating *Mycobacterium tuberculosis* (MTB) transmission within their cells and to the community at large. Transmission occurs through prison staff, visitors, and released inmates. The estimated prevalence of pulmonary tuberculosis disease in the prison systems is reported to be much higher than the average estimates in the general population, irrespective of economic status and the population TB burden of the country [23]. In European prisons, the prevalence of TB was estimated to be up to 17 times higher than in the general population [19]. A similar epidemiological situation was described in low and middle income countries, like Bangladesh, Thailand, and Ethiopia, where TB prevalence has been reported as almost four, eight, and seven times higher, respectively, among prison inmates compared to the general population [18, 20, 24]. In Ethiopia However only a small number of studies documented the prevalence of smear positive pulmonary tuberculosis among prison inmates in the country in general and in North Gondar Administrative Zone prisons in particular. We believe that the current study can serve as a baseline data to forecast the prevalence of smear positive pulmonary tuberculosis and HIV co-infection in North Gondar Administrative Zone prisons.

This study showed a prevalence of 5.3 % smear positive pulmonary tuberculosis rates among tuberculosis suspected prison inmates, making a point prevalence of 384.6 per 100,000 prison populations. Previously, the prevalence of all forms of TB in the Amhara Regional State, Ethiopia, was reported as 643 per 100,000 populations, while the prevalence of smear positive TB was 168 per 100,000 populations in the same administrative region. Therefore, the prevalence of smear positive pulmonary tuberculosis among North Gondar prison inmates was almost 2.3 times higher than previous reports in the region [25]. This indicates an increased risk of transmission of tuberculosis which could lead even to an outbreak in any of the North Gondar prisons and the general population at large, unless immediate measures are taken. However, the prevalence of smear positive pulmonary tuberculosis among tuberculosis suspected prison inmates of North Gondar was lower than what was reported from Gamo Goffa zone (19.4 %), Southern Ethiopia. The prevalence of smear positive pulmonary tuberculosis in prisons was reported as 8-fold higher than the prevalence in the general population in Southern Ethiopia [26]. The prevalence of smear positive pulmonary tuberculosis among tuberculosis suspected prison inmates of North Gondar prisons was lower than the reported cases from some other Ethiopian prisons, 1913/100,000 in eastern Ethiopia, and 629/100,000 in Southern Ethiopia [18, 26]. Furthermore, the single previous study conducted in the prison of Gondar town reported a prevalence of 1482 per 100,000 smear positive pulmonary tuberculosis [27] which was higher than that of the present study. In this study, the prevalence of smear positive pulmonary tuberculosis was determined among the TB suspected inmates of the four prisons, namely Debark, Dabat, Chilga and Gondar towns that may have its own connotations about the prevalence difference as the number of prison inmates differed from prison to prison. In the current study, the prevalence of smear positive pulmonary tuberculosis among TB suspects of the Gondar town prison was 6.7 % (*n* = 10/15).

The study revealed that 60 % of the smear positive and 81.3 % of the smear negative prison inmates developed cough after imprisonment. Longer duration of cough, transfer of prisoners from one prison to another before the completion of treatment, and diagnostic and treatment delays, could contribute to inter-prison and prison-to-population pulmonary tuberculosis transmission.

In this study, none of the smear positive prison inmates showed drug resistance against rifampicin. However, different reports showed a higher rate of MDR-TB infection among prison inmates in different parts of the world, particularly African countries [20, 28]. For example, the rates of drug resistance were significantly high among prison inmates, with rate ratios (RR) of 1.9 for MDR-TB in Bangladesh [29]. Data also showed no significant association between the duration of cough and smear positive pulmonary tuberculosis infection. However, extended time before patients get diagnosed and treated for pulmonary tuberculosis renders the smear positive cases to transmit the infection to many others. This could be intensified by the nature of the cells shared by the inmates. The presence of more than 100 prison inmates per cell was significantly associated with smear positive PTB infection (*P* < 0.01). This truly indicates that prison cells in the study area were poorly ventilated and overcrowded. Prison inmates reported that cigarette smokers were seven times more likely to develop smear positive pulmonary tuberculosis than those who did not smoked. Several studies have linked smoking with tuberculosis [30, 31], underlining that heavy smoking is associated with pulmonary tuberculosis disease.

Malnourished prison inmates were found sixteen times more likely to develop smear positive PTB compared with those who were not malnourished. The association between tuberculosis and malnutrition has been recognized for a long time. Malnutrition may predispose to TB, and in turn TB often causes malnutrition [32, 33]. In a rat model as a consequence of malnutrition, there were low numbers of alveolar macrophages (AMs) in the bronchoalveolar lavage fluid and toxic radicals such as NO releases by AMs were impaired [34].

Our study showed that smear positive pulmonary tuberculosis among TB suspected prison inmates was significantly associated with a history of contact with active TB cases (*P* = 0.035). Thirty percent of the TB suspects had a history of previous contact with active TB patients and showed clinical signs and symptoms for PTB. Of these inmates 3.9 % were smear positive. This shows that tuberculosis causing bacteria is circulating in the prisons making the risk of acquiring TB very high. On the other hand, 6 % of the TB suspects were positive for HIV, and 1.4 % of them had smear positive pulmonary tuberculosis co-infection. The prevalence of HIV infection among the TB infected inmates was 27 %. Before this report, the prevalence of HIV infection among smear positive and smear negative pulmonary tuberculosis cases was reported as 19 and 26 % in the Southern parts of Ethiopia [35]. The prevalence of TB/HIV co- infection in the current study was higher than reports from some African countries such as Tanzanian (22 %) [36]; but it was lower than that of a study conducted in northwest Ethiopia and Malawi (34.6 and 73 %, respectively) [27, 28]. A core challenge to TB control in prison systems is having to deal with the dual epidemics of HIV and TB as well as other co-infections, such as hepatitis B or C virus. Given the impact that HIV has on TB cases and vice versa, coordination between TB and HIV programs is vital [37]. However, this may be limited due to poor surveillance of HIV among prison inmates with TB, challenges in the diagnosis of TB among people living with HIV, lack of joint planning and mobilization for TB/HIV co-infection, and inadequate human resources capacity for managing the TB/HIV co-infection [38, 39]. In addition, a coordinated system supported by the ministries of health, welfare, and justice or interior should implement a holistic approach to treat TB cases in correctional facilities.

The limitation of the present study was that only the Auramine O stain microscopic examination which still has a lower sensitivity than the culture method was used.

## Conclusion

The prevalence of smear-positive pulmonary tuberculosis among North Gondar prison inmates was still high although lower than that of previous reports. There was a high prevalence of HIV infection among smear positive PTB cases. The presence of a large number of inmates per cell, smoking, malnutrition, and previous history of contact with TB cases were found to be significant risk factors for acquiring pulmonary tuberculosis in prisons. Failure to control tuberculosis in prisons has the potential to disseminate tuberculosis to the general population. Therefore, establishing a regular tuberculosis screening program requires urgent implementation. Reducing the burden of prison inmates in a particular cell, prevention of malnutrition, establishing ventilation systems can possibly minimize the transmission of tuberculosis among prison inmates. Moreover, screening prisoners for HIV infection prior to imprisonment may clarify the risk of acquiring HIV within the prison, particularly for prison inmates incarcerated for a long period of time.

## Abbreviations

AFB: Acid-fast bacilli
AMS: Alveolar macrophages
AOR: Adjusted Odds ratio
BMI: Body Mass Index
CMHS: College of Medicine and Health Sciences
FM: Fluorescence Microscope
GCMHS: Gondar College of Medicine and Health Sciences
HIV: Human immunodeficiency virus
LED: Light emitting diode
MDR-TB: Multi-drug resistant tuberculosis
MTB: *Mycobacterium tuberculosis*
MTBC: Mycobacterium tuberculosis complex
NO: Nitric oxide
NTLCP: National Tuberculosis and Leprosy control Program
PICT: Providing Initiative Counseling and Testing
PTB: Pulmonary tuberculosis
RIF: Rifampicin
SD: Standard deviation
SOPs: Standard Operational Procedures
SPSS: Statistical Package for Social Sciences
SSA: Sub-Saharan Africa
TB: Tuberculosis
UOG: University of Gondar
WHO: World Health Organization
